# High-density lipoprotein subclasses and cardiovascular disease and mortality in type 2 diabetes: analysis from the Hong Kong Diabetes Biobank

**DOI:** 10.1186/s12933-022-01726-y

**Published:** 2022-12-31

**Authors:** Qiao Jin, Eric S. H. Lau, Andrea O. Luk, Claudia H. T. Tam, Risa Ozaki, Cadmon K. P. Lim, Hongjiang Wu, Elaine Y. K. Chow, Alice P. S. Kong, Heung Man Lee, Baoqi Fan, Alex C. W. Ng, Guozhi Jiang, Ka Fai Lee, Shing Chung Siu, Grace Hui, Chiu Chi Tsang, Kam Piu Lau, Jenny Y. Leung, Man-wo Tsang, Elaine Y. N. Cheung, Grace Kam, Ip Tim Lau, June K. Li, Vincent T. Yeung, Emmy Lau, Stanley Lo, Samuel Fung, Yuk Lun Cheng, Chun Chung Chow, Weichuan Yu, Stephen K. W. Tsui, Yu Huang, Hui-yao Lan, Cheuk Chun Szeto, Wing Yee So, Alicia J. Jenkins, Juliana C. N. Chan, Ronald C. W. Ma, the Hong Kong Diabetes Biobank Study Group

**Affiliations:** 1 Department of Medicine and Therapeutics, Prince of Wales Hospital, The Chinese University of Hong Kong, Shatin, Hong Kong Special Administrative Region China; 2 Hong Kong Institute of Diabetes and Obesity, The Chinese University of Hong Kong, Shatin, Hong Kong Special Administrative Region China; 3 Li Ka Shing Institute of Health Sciences, The Chinese University of Hong Kong, Shatin, Hong Kong Special Administrative Region China; 4 CUHK-SJTU Joint Research Centre on Diabetes Genomics and Precision Medicine, Shatin, Hong Kong Special Administrative Region China; 5 School of Public Health (Shenzhen), Sun Yat-sen University, Shenzhen, Guangdong China; 6 Department of Medicine and Geriatrics, Kwong Wah Hospital, Yau Ma Tei, Hong Kong Special Administrative Region China; 7 Diabetes Centre, Tung Wah Eastern Hospital, Sheung Wan, Hong Kong Special Administrative Region China; 8 Diabetes and Education Centre, Alice Ho Miu Ling Nethersole Hospital, Tai Po, Hong Kong Special Administrative Region China; 9 North District Hospital, Sheung Shui, Hong Kong Special Administrative Region China; 10 Department of Medicine and Geriatrics, Ruttonjee Hospital, Wan Chai, Hong Kong Special Administrative Region China; 11 Department of Medicine and Geriatrics, United Christian Hospital, Kwun Tong, Hong Kong Special Administrative Region China; 12 Tseung Kwan O Hospital, Hang Hau, Hong Kong Special Administrative Region China; 13 Department of Medicine, Yan Chai Hospital, Tsuen Wan, Hong Kong Special Administrative Region China; 14 Centre for Diabetes Education and Management, Our Lady of Maryknoll Hospital, Wong Tai Sin, Hong Kong Special Administrative Region China; 15 Department of Medicine, Pamela Youde Nethersole Eastern Hospital, Chai Wan, Hong Kong Special Administrative Region China; 16 Department of Medicine and Geriatrics, Princess Margaret Hospital, Lai Chi Kok, Hong Kong Special Administrative Region China; 17 Department of Medicine, Alice Ho Miu Ling Nethersole Hospital, Tai Po, Hong Kong Special Administrative Region China; 18 Department of Electronic and Computer Engineering, The Hong Kong University of Science and Technology, Kowloon, Hong Kong Special Administrative Region China; 19 School of Biomedical Sciences, The Chinese University of Hong Kong, Shatin, Hong Kong Special Administrative Region China; 20 Department of Biomedical Sciences, City University of Hong Kong, Kowloon, Hong Kong Special Administrative Region China; 21 NHMRC Clinical Trials Centre, University of Sydney, Sydney, Australia

**Keywords:** Cardiovascular disease, High-density lipoprotein particles, High-density lipoprotein subclasses, Mortality, Prognostic marker, Residual risk, Risk stratification, Type 2 diabetes

## Abstract

**Objective:**

High-density lipoproteins (HDL) comprise particles of different size, density and composition and their vasoprotective functions may differ. Diabetes modifies the composition and function of HDL. We assessed associations of HDL size-based subclasses with incident cardiovascular disease (CVD) and mortality and their prognostic utility.

**Research design and methods:**

HDL subclasses by nuclear magnetic resonance spectroscopy were determined in sera from 1991 fasted adults with type 2 diabetes (T2D) consecutively recruited from March 2014 to February 2015 in Hong Kong. HDL was divided into small, medium, large and very large subclasses. Associations (per SD increment) with outcomes were evaluated using multivariate Cox proportional hazards models. C-statistic, integrated discrimination index (IDI), and categorial and continuous net reclassification improvement (NRI) were used to assess predictive value.

**Results:**

Over median (IQR) 5.2 (5.0–5.4) years, 125 participants developed incident CVD and 90 participants died. Small HDL particles (HDL-P) were inversely associated with incident CVD [hazard ratio (HR) 0.65 (95% CI 0.52, 0.81)] and all-cause mortality [0.47 (0.38, 0.59)] (false discovery rate < 0.05). Very large HDL-P were positively associated with all-cause mortality [1.75 (1.19, 2.58)]. Small HDL-P improved prediction of mortality [C-statistic 0.034 (0.013, 0.055), IDI 0.052 (0.014, 0.103), categorical NRI 0.156 (0.006, 0.252), and continuous NRI 0.571 (0.246, 0.851)] and CVD [IDI 0.017 (0.003, 0.038) and continuous NRI 0.282 (0.088, 0.486)] over the RECODe model.

**Conclusion:**

Small HDL-P were inversely associated with incident CVD and all-cause mortality and improved risk stratification for adverse outcomes in people with T2D. HDL-P may be used as markers for residual risk in people with T2D.

**Supplementary Information:**

The online version contains supplementary material available at 10.1186/s12933-022-01726-y.

## Introduction

Diabetes increases the risk of cardiovascular disease (CVD) by 2-to-4 fold [1] and CVD accounts for two-thirds of death in type 2 diabetes (T2D) [2]. Lowering low-density lipoprotein cholesterol (LDL-C) improves cardiovascular outcomes, however, substantial residual risk remains despite optimal LDL-C levels [3]. High-density lipoproteins (HDL) exert potential vascular protective effects and high-density lipoprotein cholesterol (HDL-C) has been consistently inversely associated with CVD in epidemiological studies. Nevertheless, Mendelian randomization analyses and clinical trials failed to support a causal role of HDL-C, suggesting that cholesterol across HDL subclasses may inadequately reflect HDL’s vasoprotective functions. HDL particles (HDL-P) vary in size, density, shape, lipid/protein composition and associated enzymes, and size-based HDL subclasses differ in function [3].

HDL may impact glucose homeostasis through pancreas and skeletal muscles and HDL-C levels have been inversely associated with incident T2D [4, 5]. The antidiabetic functionality of HDL differ by particles quantified by nuclear magnetic resonance (NMR) spectroscopy: very large and large HDL-P appeared to drive the inverse association [4, 5]. There was a progressive decrease in large HDL-P and an increase in small HDL-P in individuals with normal glucose, impaired glucose metabolism, and new diabetes [6] and large HDL-P were associated with higher insulin sensitivity and secretion [7]. The vascular function of HDL may also differ by particle size, although mixed results have been reported. Large and medium HDL-P have been inversely associated with incident cardiovascular events [8] and myocardial infarction [9] in large population-based studies using NMR, while small HDL-P have been associated with increased risk of ischemic stroke [9]. Different methods are available to assess HDL subclasses. Medium and small HDL-P as measured by high-performance liquid chromatography (HPLC) have been inversely associated with total and subtypes of stroke [10]. Smaller HDL (HDL_3_-C) cholesterol as measured by density gradient ultracentrifugation was the main determinant for the inverse association between HDL-C and incident coronary heart disease (CHD) [11]. Low small HDL-P or HDL_3_-C were found to be associated with all-cause or cardiovascular mortality using different quantification methods [12–16].

T2D also modifies the structure and composition of HDL, which may result in dysfunctional HDL [17] or even converting HDL vascular function [18]. T2D can selectively impair the ATP-binding cassette transporter A1-specific cellular cholesterol efflux capacity (ABCA1 CEC) of small HDL-P, the primary step of reverse cholesterol transport (RCT) [19]. CVD in people with long-term type 1 diabetes (T1D) was positively associated with large HDL-P and negatively with medium HDL-P by NMR [20], although the redistribution of HDL differs between T1D and T2D [6, 21]. Lipid levels and lipoprotein subclass distributions are usually adversely altered in people with diabetes, by obesity, insulin resistance, poorer glycemic control, renal dysfunction and diet [22]; hence, large studies, ideally longitudinal in nature with hard clinical events as the outcomes are desirable. Large studies with detailed subject characterization for traditional and novel risk factors, such as inflammation, will enable adjustment for covariates and confounders and robust conclusions. In the current study, we assessed the associations of NMR-determined HDL subclasses with incident CVD and all-cause mortality and their prognostic value in a well-phenotyped prospective cohort of adults with T2D.

## Research design and methods

### Participants

The Hong Kong Diabetes Biobank (HKDB) is a multicenter prospective cohort study coordinated by the Prince of Wales Hospital (PWH), the teaching hospital of the Chinese University of Hong Kong [23–25]. It is based on a similar design to the Hong Kong Diabetes Register (established at PWH since 1995), a quality-improvement program incorporating comprehensive and structured assessments of risk factors and diabetes complications and in which patients were consented for prospective follow-up and archiving of biospecimens for research purpose [26]. In 2000, the Hong Kong Hospital Authority set up a territory-wide diabetes risk assessment program via establishing hospital-based diabetes centers and adopting a similar assessment method for diabetes complications. Initiated in 2014, HKDB enrolled participants from 11 diabetes centers at major public hospitals across Hong Kong using similar enrollment and assessment methods, aiming to establish a multicenter diabetes register and biobank for biomarker discovery. All participants were invited to take part in the study when attending a scheduled and standardized diabetes complications assessment and the recruitment methods, collection of anthropometric, lifestyle factors, biochemical investigations, and blood samples for research purpose have been detailed previously [23–25]. Once enrolled, participants will be followed till death. All participants provided written informed consent at the time of enrolment and the study was approved by the Joint Chinese University of Hong Kong-New Territories East Cluster Clinical Research Ethics Committee, as well as the Clinical Research Ethics Committee of each participating hospital.

### Demographic and laboratory measurements

Demographic data, medical and medication history were documented via face-to-face interview based on standardized questionnaires. Blood pressure was measured in both arms after at least 5-min sitting and a mean value was used for analysis. Body mass index (BMI) was calculated as weight in kilograms divided by height in meters squared. Blood samples after at least 8-hour overnight fasting were measured for glycated hemoglobin (HbA_1C_), serum creatinine, and the traditional lipid profile (total cholesterol, HDL-C, triglycerides, and calculated LDL-C). A random spot urine sample was measured for urinary albumin. The Chronic Kidney Disease Epidemiology Collaboration equation was used for estimated glomerular filtration rate (eGFR) calculation [27].

### HDL subclasses

Sera from fasted participants were stored (− 80 °C) at PWH prior to quantification. Concentrations of total, very large (average diameter 14.3 nm), large (12.1 nm), medium (10.9 nm), and small (8.7 nm) HDL-P as well as mean HDL-P size were quantified using an automated NMR spectroscopy platform (Nightingale Health, Helsinki, Finland). This NMR platform has been extensively applied in large-scale epidemiological studies [28, 29]. Measurements of HDL subclasses have been calibrated using HPLC and the detailed experimentation has been described previously [28]. The coefficients of variation (CV%) were below 5% for lipoprotein subclasses [30]. Serum samples consecutively collected from March 2014 to February 2015 (N = 2000) were quantified. Two samples failed quality control and seven non-T2D participants were excluded. Consequently, 1991 participants were included in the analysis (Additional file 1: Fig. S1). Zero values in HDL measures (3.1% for very large HDL-P, 5.1% for large HDL-P, and 0.3% for medium HDL-P) indicating levels below the detection limit were replaced with half of the minimum value in the corresponding measurement.

### Outcomes

Incident CVD was defined by discharge codes based on the International Classification of Diseases, Ninth Revision (ICD-9), retrieved from the electronic medical records system set up by the Hong Kong Hospital Authority. CVD was defined as the first occurrence of cardiovascular death, CHD (myocardial infarction, ischemic heart disease, or angina pectoris), stroke (ischemic stroke except transient ischemic attack or hemorrhagic stroke), peripheral vascular disease (amputation, gangrene, or peripheral revascularization), or hospitalization for heart failure [24]. Vital status was retrieved from the Hong Kong Death Registry and specific cause of death was defined by ICD-10 codes (I00–I99 for cardiovascular death).

### Statistical analysis

Variables were presented as mean ± standard deviation (SD), median (interquartile range [IQR]), or number (%) and compared by t-test, Wilcoxon rank sum test, or Chi-square test as appropriate. Intercorrelations between HDL subclasses were assessed using Pearson correlation test. Cumulative incidences of incident CVD and all-cause mortality were estimated with the Kaplan-Meier method and differences across HDL-P tertiles were compared by log-rank tests.

The associations of HDL subclasses in tertiles (the lowest tertile as the reference group) with time to incident CVD and all-cause mortality were examined using Cox proportional hazard regressions with sequential adjustment for potential confounders nominally associated with the outcomes (*P* < 0.10) in the univariate comparison (Table 1) or selected for biological plausibility (e.g. lipid-lowering drugs and glycoprotein acetyls [a validated systemic inflammatory marker by NMR] [31, 32]). Models constructed included the unadjusted model (model 1) and models adjusted for age and sex (model 2), model 2 plus smoking, diabetes duration, systolic blood pressure (SBP), BMI, HbA_1C_, eGFR, ln [urinary albumin-creatinine ratio (UACR)], and glycoprotein acetyls (model 3), model 3 plus use of oral anti-hyperglycemic drugs, insulin use, anti-hypertensive drugs, renin-angiotensin system (RAS) blockers, and lipid-lowering drugs (model 4), model 4 plus LDL-C (model 5), model 5 plus ln (triglycerides) (model 6), and model 6 plus HDL-C (the fully adjusted model, model 7). For all-cause mortality, prevalent CVD was added in model 3.

**Table 1:** Baseline characteristics according to incident CVD and death

Variable	Overall N = 1991	Incident CVD			*P* value	Death		*P* value
		No, N = 1322		Yes, N = 125		No, N = 1901	Yes, N = 90	
Demographic								
Age, years	61.1 ± 11.0	58.9 ± 10.9	63.6 ± 11.1		**< 0.0001**	60.7 ± 10.9	69.6 ± 9.9	**< 0.0001**
Male gender	1189 (59.7)	728 (55.1)	87 (70.0)		**0.002**	2087 (56.1)	66 (76.7)	**0.002**
Smoking status, ever	672 (33.8)	369 (27.9)	58 (46.4)		**< 0.0001**	1082 (29.1)	41 (47.7)	**< 0.0001**
Clinical and laboratory								
Diabetes duration, years	11.4 ± 8.7	10.3 ± 8.3	14.2 ± 9.0		**< 0.0001**	11.2 ± 8.6	15.3 ± 10.4	**0.0004**
SBP, mmHg	135.5 ± 18.4	132.5 ± 17.6	141.4 ± 19.0		**< 0.0001**	135.3 ± 18.2	139.7 ± 21.4	0.06
BMI, kg/m^2^	26.5 ± 4.6	26.2 ± 4.5	27.2 ± 5.2		0.05	26.5 ± 4.6	26.1 ± 5.4	0.50
HbA_1C_, %	7.5 ± 1.4	7.5 ± 1.4	8.0 ± 1.7		**0.001**	7.5 ± 1.4	7.8 ± 1.5	0.14
eGFR, mL/min/1.73 m^2^	75.8 ± 26.4	81.6 ± 24.1	64.5 ± 29.8		**< 0.0001**	77.0 ± 25.6	49.7 ± 30.2	**< 0.0001**
UACR, mg/mmol	2.7 (0.7–17.4)	1.8 (0.6-9.0)	16.4 (1.7-187.7)		**< 0.0001**	2.4 (0.7–15.0)	20.7 (3.6-200.7)	**< 0.0001**
Triglycerides, mmol/L	1.3 (1.0–2.0)	1.3 (0.9–1.9)	1.6 (1.0-2.3)		**0.001**	1.4 (1.0–2.0)	1.3 (1.0-2.1)	0.74
TC, mmol/L	4.4 ± 1.0	4.4 ± 0.9	4.7 ± 1.1		**0.01**	4.4 ± 0.9	4.3 ± 1.2	0.93
HDL-C, mmol/L	1.3 ± 0.4	1.3 ± 0.4	1.2 ± 0.4		**0.001**	1.3 ± 0.4	1.2 ± 0.3	**0.001**
LDL-C, mmol/L	2.3 ± 0.8	2.4 ± 0.8	2.7 ± 1.0		**0.004**	2.3 ± 0.8	2.4 ± 1.0	0.42
Glycoprotein acetyls, mmol/L	1.1 ± 0.2	1.1 ± 0.2	1.2 ± 0.2		**< 0.0001**	1.1 ± 0.2	1.2 ± 0.2	**0.02**
Total HDL-P, µmol/L	21.6 ± 3.1	22.1 ± 3.1	20.8 ± 3.1		**< 0.0001**	21.7 ± 3.1	19.5 ± 2.7	**< 0.0001**
Very large HDL-P, µmol/L	0.26 ± 0.11	0.26 ± 0.12	0.28 ± 0.11		0.18	0.26 ± 0.11	0.32 ± 0.14	**0.0001**
Large HDL-P, µmol/L	1.4 ± 0.9	1.5 ± 1.0	1.3 ± 0.9		0.08	1.4 ± 0.9	1.6 ± 0.9	0.07
Medium HDL-P, µmol/L	4.9 ± 1.2	5.1 ± 1.2	4.6 ± 1.3		**0.0001**	5.0 ± 1.2	4.4 ± 1.0	**< 0.0001**
Small HDL-P, µmol/L	15.0 ± 1.9	15.2 ± 1.9	14.5 ± 1.8		**0.0001**	15.1 ± 1.8	13.2 ± 2.0	**< 0.0001**
HDL-P size, nm	9.5 ± 0.2	9.5 ± 0.2	9.5 ± 0.2		0.29	9.5 ± 0.2	9.6 ± 0.2	**0.002**
Medical history								
Diabetic retinopathy	517 (26.0)	315 (23.8)	43 (34.4)		**0.0004**	482 (25.4)	35 (38.9)	**0.0004**
Cardiovascular disease	544 (27.3)	NA	NA		NA	501 (26.4)	43 (47.8)	**< 0.0001**
Medication history								
Oral antihyperglycemic drugs	1682 (84.5)	1154 (87.3)	109 (87.2)		0.89	1626 (85.5)	56 (62.2)	**< 0.0001**
Insulin	753 (37.8)	461 (34.9)	64 (51.2)		**0.001**	701 (36.9)	52 (57.8)	**0.0001**
Lipid lowering drugs	1360 (68.3)	810 (61.3)	76 (60.8)		0.89	1304 (68.6)	56 (62.2)	0.45
Statins	1333 (67.0)	786 (59.5)	75 (60.0)		0.99	1279 (67.3)	54 (60.0)	0.22
Fibrates	80 (4.0)	55 (4.2)	6 (4.8)		0.92	77 (4.0)	3 (3.3)	0.96
Antihypertensive drugs	1524 (76.5)	909 (68.8)	109 (87.2)		**< 0.0001**	1448 (76.2)	76 (84.4)	0.08
RAS blockers	1183 (59.4)	708 (53.6)	87 (69.6)		**0.001**	1279 (67.3)	55 (61.1)	0.75

Data are presented as mean ± SD, number (percentage), or median (interquartile range)

*BMI* body mass index; *CVD* cardiovascular disease; *eGFR* estimated glomerular filtration rate; *HbA*_1C_ glycated hemoglobin; HDL-C, high-density lipoprotein cholesterol; *HDL-P* high-density lipoprotein particles; *LDL-C* low-density lipoprotein cholesterol; *RAS* renin-angiotensin system; *SBP* systolic blood pressure; *SD* standard deviation; *TC* Total cholesterol; *UACR* urinary albumin-creatinine ratio

*P* values for differences between events were obtained by t-test, Chi-squared test, or Wilcoxon rank sum test as appropriate

The associations were further assessed per SD increment in HDL subclasses and all HDL measurements were log_e_-transformed prior to z-scaling [29]. Non-linearity of HDL subclasses with outcomes was examined with restricted cubic splines adjusting for covariates in the full model and three knots were located at the 10th, 50th, and 90th percentiles of HDL subclasses, with the median as the reference group. False discovery rate (FDR) < 0.05 was considered significant to account for multiple testing [33].

The prognostic value of HDL subclasses was assessed by C-statistic, integrated discrimination improvement (IDI), categorical and continuous net reclassification improvement (NRI) over the Risk Equations for Complications of Type 2 Diabetes (RECODe) [34]. The calculation of IDI and NRI were based on 5-year risk and the risk categories for categorical NRI were < 5%, 5–10%, and > 10%. Bootstrapping with 1000 iterations were used to estimate the 95% confidence interval (CI). Multiple imputation was not performed as missing values were few (≤ 2%) for each covariate. All analyses were performed in R version 4.0.3.

### Sensitivity analyses

To account for the competing risk of non-cardiovascular death, competing risk regression proposed by Fine and Gray was used to estimate the sub-distribution hazard ratio (HR) with 95% confidence interval (CI) for incident CVD [35]. To assess the robustness of the associations, we repeated the analyses after excluding participants with missing HDL-C measurements (N = 110).

## Results

### Baseline characteristics

Table 1 summarizes the baseline characteristics of the study population. Mean age was 61.1 years; 59.7% were male; and mean known diabetes duration was 11.4 years. All HDL-related measurements were significantly correlated with each other, with correlation coefficients ranging from − 0.30 to 0.97 (Additional file 1: Table S1, *P* < 0.001). While very large, large, and medium HDL-P were mutually positively correlated, small HDL-P were negatively correlated with very large and large HDL-P and positively correlated with medium HDL-P.

### Association with incident CVD

Among 1447 participants free of prevalent CVD at baseline, 125 (8.6%) participants developed incident CVD during a median (IQR) follow-up of 5.2 (5.0-5.4) years, corresponding to an incidence rate (95% CI) of 17.5 (14.6, 20.9) per 1000-person years. Baseline characteristics of participants by incident CVD are presented in Table 1. Participants who developed incident CVD tended to be older, had longer diabetes duration with a male prepondence. They were more likely to be smokers and had diabetic retinopathy, with higher proportion of them using insulin, antihypertensive drugs, and RAS blockers. Participants with CVD also had higher levels of systolic blood pressure (SBP), HbA_1C_, UACR, triglycerides, total cholesterol, LDL-C, and glycoprotein acetyls and had lower levels of eGFR, HDL-C, total, medium and small HDL-P than those without.

**Fig. 1:**
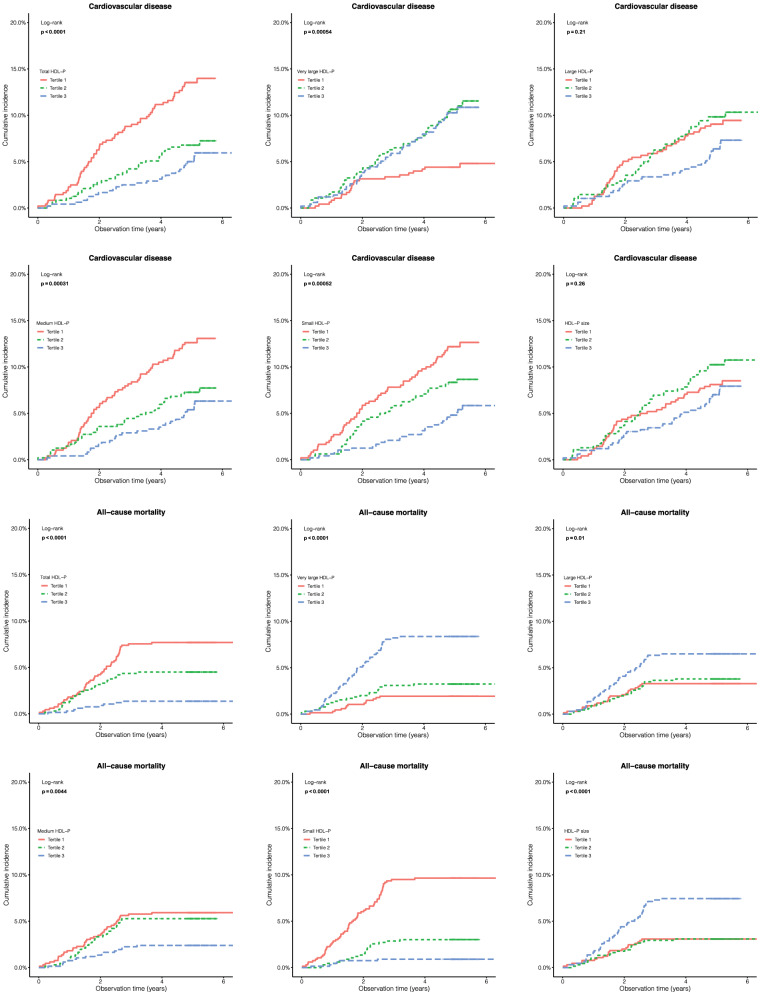
Kaplan-Meier plots for cumulative incidence of incident CVD and all-cause mortality according to tertiles of HDL-P measurements. Adjusted for age, sex, smoking, diabetes duration, systolic blood pressure, body mass index, glycated hemoglobin, estimated glomerular filtration rate, ln (urinary albumin-creatinine ratio), ln (triglycerides), low-density lipoprotein cholesterol, high-density lipoprotein cholesterol, glycoprotein acetyls, oral antihyperglycemic drugs, insulin use, renin-angiotensin system blockers, antihypertensive drugs, lipid-lowering drugs, and history of cardiovascular disease (for all-cause mortality). *P* value was obtained by the log-rank test

When categorized into tertiles (Additional file 1: Table S2) and compared to the lowest tertile, higher tertiles of, total HDL-P [HR 0.49 (95% CI 0.32, 0.74) for T2 and 0.39 (0.25, 0.60) for T3] were associated with longer time to incident CVD; higher tertiles of very large HDL-P [2.47 (1.50, 4.07) for T2 and 2.36 (1.43, 3.88) for T3] were associated with shorter time to incident CVD; higher tertiles of medium HDL-P [0.56 (0.37, 0.85) for T2 and 0.44 (0.29, 0.69) for T3], as well as small HDL-P [0.41 (0.26, 0.66) for T3] (Table 2; Fig. 1) were associated with longer time to incident CVD. Very large and small HDL-P remained significant across models adjusting for confounders, (Table 2 and Additional file 1: Table S3).

**Table 2:** Associations of HDL-P measurements with incident CVD and all-cause mortality

Variable	Incident CVD				All-cause mortality			
	Unadjusted HR (95% CI)	*P* value	Adjusted HR (95% CI)	*P* value	Unadjusted HR (95% CI)	*P* value	Adjusted HR (95% CI)	*P* value
Total HDL-P, per SD	0.64 (0.53, 0.76)	**< 0.0001**	0.71 (0.52, 0.97)	**0.03**	0.49 (0.40, 0.60)	**< 0.0001**	0.61 (0.43, 0.86)	**0.01**
First tertile	Reference		Reference		Reference		Reference	
Second tertile	0.49 (0.32, 0.74)	**0.001**	0.59 (0.36, 0.96)	**0.03**	0.58 (0.37, 0.91)	**0.02**	1.01 (0.58, 1.74)	0.99
Third tertile	0.39 (0.25, 0.60)	**< 0.0001**	0.47 (0.23, 0.95)	**0.04**	0.17 (0.08, 0.35)	**< 0.0001**	0.46 (0.18, 1.22)	0.12
Very large HDL-P, per SD	1.17 (0.96, 1.42)	0.19	1.44 (1.11, 1.87)	**0.01**	1.54 (1.19, 1.99)	**0.001**	1.75 (1.19, 2.58)	**0.01**
First tertile	Reference		Reference		Reference		Reference	
Second tertile	2.47 (1.50, 4.07)	**0.0004**	3.06 (1.79, 5.24)	**< 0.0001**	1.69 (0.85, 3.38)	0.14	1.49 (0.72, 3.11)	0.28
Third tertile	2.36 (1.43, 3.88)	**0.001**	3.23 (1.78, 5.85)	**0.0001**	4.48 (2.45, 8.20)	**< 0.0001**	4.37 (2.16, 8.84)	**< 0.0001**
Large HDL-P, per SD	0.91 (0.77, 1.07)	0.28	1.49 (1.13, 1.96)	**0.01**	1.15 (0.91, 1.45)	0.24	1.85 (1.23, 2.76)	**0.01**
First tertile	Reference		Reference		Reference		Reference	
Second tertile	1.08 (0.72, 1.63)	0.70	1.85 (1.13, 3.03)	**0.02**	1.15 (0.65, 2.04)	0.63	1.89 (0.97, 3.68)	0.06
Third tertile	0.74 (0.47, 1.16)	0.19	2.40 (1.14, 5.03)	**0.02**	2.00 (1.20, 3.35)	**0.01**	7.65 (3.52, 16.65)	**< 0.0001**
Medium HDL-P, per SD	0.70 (0.61, 0.81)	**< 0.0001**	0.95 (0.72, 1.23)	0.68	0.67 (0.58, 0.78)	**< 0.0001**	0.89 (0.66, 1.19)	0.42
First tertile	Reference		Reference		Reference		Reference	
Second tertile	0.56 (0.37, 0.85)	**0.01**	0.83 (0.50, 1.37)	0.45	0.91 (0.58, 1.42)	0.67	1.64 (0.95, 2.84)	0.08
Third tertile	0.44 (0.29, 0.69)	**0.0003**	1.11 (0.53, 2.33)	0.79	0.37 (0.21, 0.68)	**0.001**	1.66 (0.68, 4.04)	0.27
Small HDL-P, per SD	0.70 (0.60, 0.82)	**< 0.0001**	0.65 (0.52, 0.81)	**0.001**	0.51 (0.45, 0.57)	**< 0.0001**	0.47 (0.38, 0.59)	**< 0.0001**
First tertile	Reference		Reference		Reference		Reference	
Second tertile	0.67 (0.45, 1.00)	0.05	0.64 (0.40, 1.01)	0.05	0.30 (0.18, 0.50)	**< 0.0001**	0.45 (0.26, 0.79)	**0.01**
Third tertile	0.41 (0.26, 0.66)	**0.0002**	0.39 (0.22, 0.72)	**0.002**	0.09 (0.04, 0.21)	**< 0.0001**	0.18 (0.07, 0.46)	**0.0003**
HDL-P size, per SD	0.91 (0.76, 1.09)	0.33	1.68 (1.28, 2.22)	**0.001**	1.43 (1.20, 1.70)	**< 0.0001**	2.93 (2.22, 3.87)	**< 0.0001**
First tertile	Reference		Reference		Reference		Reference	
Second tertile	1.27 (0.83, 1.93)	0.27	2.12 (1.29, 3.48)	**0.003**	1.00 (0.54, 1.85)	0.99	1.51 (0.76, 3.01)	0.24
Third tertile	0.90 (0.58, 1.41)	0.65	2.63 (1.35, 5.12)	**0.005**	2.46 (1.46, 4.14)	**0.001**	5.87 (2.84, 12.15)	**< 0.0001**

All HDL-P measurements were log_e_-transformed before z-scaling. *P* values for SD change were false discovery rate corrected

*CI* confidence interval; *CVD* cardiovascular disease; *HDL-P* high-density lipoprotein particles; *HR* hazard ratio; *SD* standard deviation

Model adjusted for age, sex, smoking, diabetes duration, systolic blood pressure, body mass index, glycated hemoglobin, estimated glomerular filtration rate, ln (urinary albumin-creatinine ratio), ln (triglycerides), low-density lipoprotein cholesterol, high-density lipoprotein cholesterol, glycoprotein acetyls, oral antihyperglycemic drugs, insulin use, renin-angiotensin system blockers, antihypertensive drugs, lipid-lowering drugs, and prevalent CVD (for all-cause mortality)

**Fig. 2:**
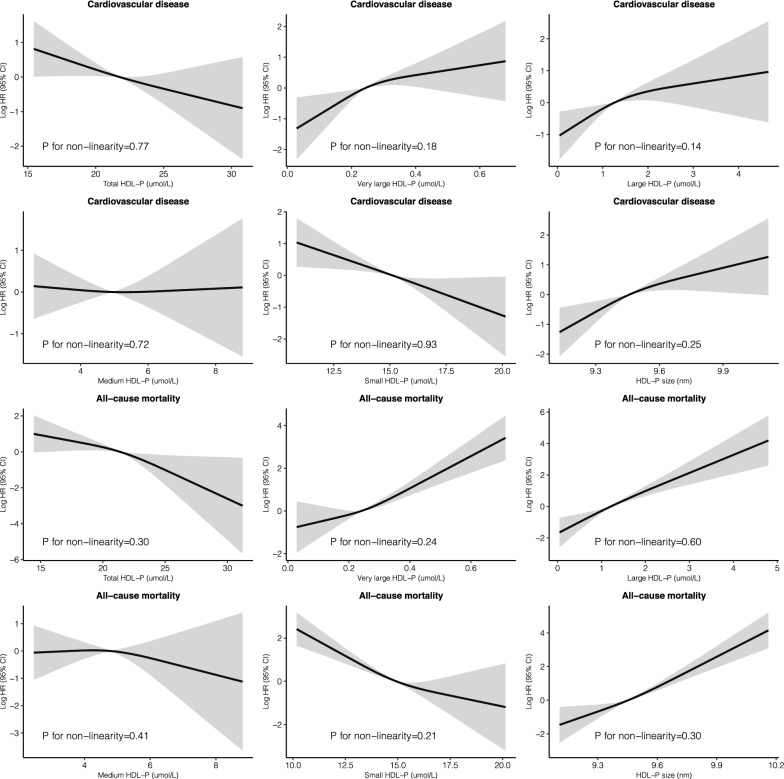
Adjusted restricted cubic spline analysis for the associations between HDL-P measurements and incident CVD and all-cause mortality. The solid lines are the adjusted log hazard ratios, and the shaded areas are 95% CIs derived from corresponding restricted cubic spline regressions. A knot was located at 10th, 50th (reference value), 90th percentiles for each of the HDL-P measurements. Analyses were adjusted for age, sex, smoking, diabetes duration, systolic blood pressure, body mass index, glycated hemoglobin, estimated glomerular filtration rate, ln (urinary albumin-creatinine ratio), ln (triglycerides), low-density lipoprotein cholesterol, high-density lipoprotein cholesterol, glycoprotein acetyls, oral anti-hyperglycemic drugs, insulin use, renin-angiotensin system blockers, anti-hypertensive drugs, lipid-lowering drugs, and history of cardiovascular disease (for all-cause mortality)

Multivariate-adjusted restricted cubic splines indicated no evidence of nonlinearity between HDL subclasses and incident CVD (*P* ranged from 0.14 to 0.93, Fig. 2). For per SD increment in HDL measurements, total HDL-P [0.64 (0.53, 0.76)], medium HDL-P [0.70 (0.61, 0.81)], and small HDL-P [0.70 (0.60, 0.82)] were associated with incident CVD, with FDR < 0.0001 (Table 2). Across models adjusting for confounders, small HDL-P remained significant (FDR = 0.001; Table 2).

### Association with all-cause mortality

A total of 90 (4.5%) participants died during follow-up, corresponding to a mortality rate of 8.9 (7.2, 11.0) per 1000-person years. The baseline characteristics are described in Table 1. Briefly, participants who died tended to be older, had longer diabetes duration, higher levels of glycoprotein acetyls, and very large HDL-P, and lower levels of medium and small HDL-P, and higher proportions of males, smokers, and prevalent diabetic complications.

When modelled in tertiles (Additional file 1: Table S2), total HDL-P [0.17 (0.08, 0.35) for T3], very large HDL-P [4.48 (2.45, 8.20) for T3], large HDL-P [2.00 (1.20, 3.35) for T3], medium HDL-P [0.37 (0.21, 0.68) for T3], small HDL-P [0.30 (0.18, 0.50) for T2 and 0.09 (0.04, 0.21) for T3], and HDL-P size [2.46 (1.46, 4.14) for T3] were associated with time to death (Table 2; Fig. 1). Very large HDL-P, large HDL-P, small HDL-P, and HDL-P size remained significant across models adjusting for key confounders (Table 2 and Additional file 1: Table S4).

Multivariable-adjusted restricted cubic splines suggested no evidence of nonlinearity between HDL subclasses and mortality (*P* ranged from 0.21 to 0.60, Fig. 2). For per SD increment in HDL measurements, total HDL-P [0.49 (0.40, 0.60)], very large HDL-P [1.54 (1.19, 1.99)], medium HDL-P [0.67 (0.58, 0.78)], small HDL-P [0.51 (0.45, 0.57)], and HDL-P size [1.43 (1.20, 1.70)] were associated with time to death, with FDR ≤ 0.001 (Table 2). Total HDL-P, very large HDL-P, small HDL-P, and HDL-P size remained significant after adjusting for confounders (Table 2 and Additional file 1: Table S4).

### Prognostic value of HDL-P subclasses

Since only small HDL-P were robustly associated with lower risk of incident CVD and all-cause mortality analyzed by tertiles or SD (Table 2), we further assessed its predictive value compared to other prognostic tools. When added to the RECODe model, small HDL-P increased the C-statistic (95% CI) from 0.829 (0.789, 0.868) to 0.863 (0.826, 0.900) with an improvement in C-statistic of 0.034 (0.013, 0.055) for all-cause mortality risk prediction (Table 3). The addition significantly increased IDI (0.052 [0.014, 0.103]), categorical (0.156 [0.006, 0.252]) and continuous NRI [0.571 (0.246, 0.851)]. Small HDL-P also increased the predictive value for incident CVD [IDI 0.017 (0.003, 0.038) and continuous NRI 0.282 (0.088, 0.486)].

**Table 3:** Predictive performances of small HDL-P for CVD and all-cause mortality over RECODe model

	Incident CVD	All-cause mortality
RECODe model, C-statistic (95% CI)	0.760 (0.715, 0.805)	0.829 (0.789, 0.868)
+ Small HDL-P, C-statistic (95% CI)	0.770 (0.726, 0.813)	0.863 (0.826, 0.900)
Change in C-statistic (95% CI)	0.010 (− 0.001, 0.029)	0.034 (0.013, 0.055)
IDI for 5-year risk (95% CI)	0.017 (0.003, 0.038)	0.052 (0.014, 0.103)
Categorical NRI for 5-year risk (95% CI)		
Case	− 0.041 (− 0.056, 0.087)	0.118 (− 0.026, 0.208)
Noncase	0.026 (− 0.009, 0.058)	0.038 (0.013, 0.067)
Overall	− 0.015 (− 0.047, 0.126)	0.156 (0.006, 0.252)
Continuous NRI for 5-year risk (95% CI)		
Case	0.183 (0.052, 0.340)	0.224 (0.034, 0.421)
Non-case	0.099 (− 0.006, 0.205)	0.348 (0.163, 0.485)
Overall	0.282 (0.088, 0.486)	0.571 (0.246, 0.851)

95% CI estimated by 1000 times bootstrapping

Risk cutoffs for categorical NRI: 5% and 10%

RECODe model: age, sex, current smoking, systolic blood pressure, glycated hemoglobin, total cholesterol, high-density lipoprotein cholesterol, estimated glomerular filtration rate, ln (urinary albumin-creatinine ratio), antihypertensive drugs, statins and CVD history (for all-cause mortality)

*CI* confidence interval; *CVD* cardiovascular disease; *HDL-P* high-density lipoprotein particles; *IDI* integrated discrimination improvement; *NRI* net reclassification improvement

### Sensitivity analyses

After accounting for the competing risk of non-cardiovascular death (31 participants died before developing incident CVD), only small HDL-P were associated with incident CVD at FDR < 0.05 (Additional file 1: Table S5). Among individuals with complete HDL measurements total and small HDL-P were inversely associated with incident CVD (N = 1370) and all-cause mortality (N = 1881); very large and large HDL-P were positively associated with all-cause mortality (FDR < 0.05; Additional file 1: Table S6).

## Discussion

In a longitudinal study of 1991 adults with T2D and a median follow-up of 5.2 years we demonstrated that NMR-determined HDL subclasses at baseline were associated with incident CVD and all-cause mortality after adjusting for confounders. Small HDL-P were consistently negatively associated with CVD and all-cause mortality and improved risk prediction beyond traditional risk factors. Total HDL-P and very large HDL-P were positively associated with all-cause mortality.

NMR-determined HDL-P have been inversely associated with CVD in a pooled study of four large (N = 15,784) cohorts [36] and with mortality in individuals undergoing cardiac catheterization [13, 14]. The association was stronger than that of HDL-C and remained significant after adjustment for HDL-C, although HDL-C was not related with CVD after adjustment for HDL-P [36]. The latter observation may relate to the fact that HDL comprises subclasses of varying size, structure, composition and functions [3]. The anti-diabetic property of HDL appeared to differ between NMR-determined subclasses, with larger HDL-P mainly inversely associated with T2D [4, 5]. The association of HDL with adverse outcomes also appeared different between subclasses and between primary and secondary prevention of CVD.

There are variable reports on the risk association between HDL-P and CVD which may be confounded by study design, population and methodology. In the China Kadoorie Biobank, different associations of HDL-P with myocardial infarction have been reported using a nested case-control cohort (N = 2378) from the general population, including around 10% with diabetes, and the inverse association was limited to larger and medium HDL-P [9]. Moreover, HDL subclasses exhibited different associations with ischemic stroke: despite an inverse association of large HDL-P, small HDL-P were positively associated with ischemic stroke and HDL-P were not associated with intracerebral hemorrhage [9]. With a larger sample size (N = 7256), large and medium HDL-P were inversely associated with cardiovascular events (myocardial infarction, ischemic stroke, cardiac revascularization, or unstable angina) in the FINRISK study in the general population (5.4% with diabetes) and no significant associations were found for small HDL-P [8].

In this study of patients with T2D with detailed phenotypes for adjustment and using the same NMR platform as that used in the FinRISK cohort, we found inverse associations of small HDL-P with CVD and all-cause mortality. Our results are supported by other reports. In a nested case-control study from the Veterans Affairs High-Density Lipoprotein Intervention Trial (N = 1061, 30.4% with diabetes), NMR-determined small HDL-P appeared to drive the inverse association of HDL-P with myocardial infarction or cardiac death. The association was consistent in individuals treated with placebo or the fibrate gemfibrozil [37]. In a study from Japan (N = 12,804, around 10% with diabetes) where HDL subclasses were measured by HPLC, medium and small HDL-P were inversely associated with total stroke and its subtypes, including ischemic and hemorrhagic stroke. There was no association of stroke with large HDL-P, although the odds ratio was generally greater than 1[10]. Using density gradient ultracentrifugation to measure cholesterol in two major HDL subfractions (larger, more buoyant HDL_2_ and smaller, denser HDL_3_), analysis combining the Jackson Heart Study (N = 4114, 16% with diabetes) and the Framingham Offspring Cohort Studies (N = 818, 7% with diabetes) found that HDL_3_-C was inversely associated with CHD while HDL_2_-C was not associated with the outcome [11]. In addition, small HDL-P appeared to be consistently inversely associated with adverse outcomes in secondary prevention populations using different quantification methods. Lower HDL_3_-C levels were consistently associated with all-cause mortality in people with myocardial infarction and mortality or myocardial infarction in people undergoing angiography, although HDL_2_-C was not significantly associated [12]. In The CATHeterization GENetics cohort (N = 3992, 28.8% with diabetes), medium and small HDL-P quantified by NMR were inversely related with all-cause mortality [38]. In a subcohort of people with or without heart failure, small HDL-P were associated with all-cause mortality or a composite outcome of mortality and major adverse cardiovascular events [13]. In the LUdwigshafen RIsk and Cardiovascular Health Cohort including patients referred for coronary angiography (N = 2290, 40.1% with diabetes), small HDL-P were also found to primarily mediate the inverse association of HDL-P with cardiovascular mortality [14].

Our result that small HDL-P levels at baseline were inversely associated with all-cause mortality is consistent with previous studies [12–14, 38] and we also found an inverse association between small HDL-P and CVD, which contrasts with prior studies using the same NMR platform [8, 9]. Different study population may explain these differences. Prior studies were population-based with few individuals (< 10%) having diabetes or dyslipidemia[8, 9]. In our study, all patients had T2D with older age and long duration of diabetes (11 years), putting them at high risk of CVD and death. The inverse association of small HDL-P with adverse outcomes in people at risk of or suspected of CVD [12, 13] was in line with our findings. Less small and medium HDL-P have been associated with CVD in a structure-function study [39]. In our study, we also found inverse association of medium HDL-P with CVD and mortality, although this was rendered non-significant after different adjustments. Small and medium HDL-P contribute to > 80% of circulating HDL-P [29] which might explain their large contribution to the inverse association of HDL-P with CVD [36]. A recent multivariate Mendelian randomization study suggested a potential protective effect of small and medium HDL-P for CVD, although the effect size of small HDL-P was smaller than that of medium HDL-P [40]. Mechanistically, small HDL-P play a central role in RCT by initiating ABCA1 CEC, which is one of HDL’s primary anti-atherogenic functions [41]. Diabetes selectively impairs small HDL-P related CEC [19], which may partly explain the increased CVD risk in people with diabetes. Small HDL-P also have greater anti-inflammatory, anti-oxidant, and endothelial function protection capacity than larger HDL subclasses [42, 43].

NMR-determined larger HDL-P have been inversely associated with hyperglycemia or diabetes [4, 5] and CVD in previous studies [8, 9]. Contrastingly, very large HDL-P were associated with higher risk of all-cause mortality consistently and with CVD in some models in the current study in T2D. Larger HDL-P contribute substantially to HDL-C levels [29], however, genetically predicted HDL-C across HDL subclasses has not been causally associated with CVD in Mendelian randomization analysis and significantly increased HDL-C levels by cholesteryl ester transfer protein (CETP) inhibitors with reduction in small HDL-P levels failed to translate into cardiovascular benefits [3]. Chronic inflammatory states, including diabetes, can alter HDL composition and function [17, 18, 44]. Instead of a stimulatory effect in healthy subjects, HDL from people with diabetes tended to inhibit endothelial cell release of nitric oxide [18], leading to endothelial dysfunction and impaired vasodilation. Also, large HDL-P may become pro-inflammatory and impair its anti-oxidant functions; pro-oxidant/pro-inflammatory HDL-P were associated with increased risk of acute coronary syndrome in people at high CVD risk [45] and HDL-P size has been associated with increased risk of CVD in a multivariate Mendelian randomization analysis [40]. Regarding heart failure, increased large HDL-P and decreased small HDL-P exist in people with heart failure with reduced ejection fraction compared with those without heart failure. Furthermore, in this study higher large HDL-P levels were positively associated with all-cause mortality in people with heart failure with preserved ejection fraction or without heart failure [13]. One possible explanation of the observation in our study is that very large HDL-P may be enriched with dysfunctional HDL, which are more pro-inflammatory and pro-oxidant in people with diabetes and comorbidities (e.g., chronic kidney disease) [17, 18, 46]. More studies are warranted to explore HDL remodeling (size, proteome, and lipidome) between individuals with or without diabetes and their relationships with clinical outcomes.

HDL-C remains an important prognostic marker recommended by many clinical guidelines, and incorporated into risk equations [34]. In T2D adults we found small HDL-P were associated with CVD and all-cause mortality both with and without adjustment for HDL-C. We further assessed the prognostic value of small HDL-P beyond traditional risk factors and found that small HDL-P showed incremental predictive value over a well-established prediction model (RECODe), suggesting that HDL subclasses may be an important marker for residual risk in diabetes populations. Despite relatively limited numbers of death (N = 90) and a C-statistic of 0.83 by the RECODe model, small HDL-P significantly improved the C-statistic (from 0.83 to 0.86), IDI, and categorical and continuous NRI for all-cause mortality risk. Small HDL-P also improved IDI and continuous NRI for CVD prediction. These positive results support the need for even larger studies with longer follow-up periods of the prognostic value of small HDL-P.

Larger and small HDL-P seem consistently inversely and positively (respectively) associated with diabetes in different studies [4, 5, 7, 39], however, for HDL-Ps associations with CVD and mortality, mixed results have been reported in different study settings using different methods [8, 9, 12, 13]. Diabetes and its underlying chronic inflammatory state can modify HDL subclass redistribution and function [3]. To our knowledge, the current study is the first to examine the association of HDL subclasses with incident CVD and all-cause mortality in a T2D population, and we further assessed the prognostic significance of HDL subfractions [47, 48]. Other key strengths include dense phenotyping facilitating comprehensive adjustment, a well-validated quantification platform with stringent quality control, and consistent results in tertile and SD change (FDR corrected) and across sensitivity analyses, all contributing to the robustness of our findings. Nevertheless, there are several limitations. All participants were Chinese and independent data for replication were unavailable, limiting the generalizability of our findings. The improvement in prognosis was small when adding small HDL-P over conventional risk equations, which might be due to the relatively limited number of events and larger and longer studies are warranted. Although the current results suggest prognostic potential of small HDL-P, whether the measurement of HDL-P should be implemented in clinical practice need to be further assessed in cost-effective studies. Over half of the participants (67%) were on statins and 80 (4%) participants were on fibrates at baseline, which may influence lipoprotein metabolism. However, lipid lowering drugs (including statins) have been accounted for in all data analyses. HDL subclasses were measured by NMR spectroscopy and HDL_2_-C and HDL_3_-C levels were not available, hence results in the current study may not be directly comparable with findings using other quantification methods [49]. The associations were observational and residual confounding cannot be ruled out. The vascular functions of different HDL-P in diabetes need further evaluation in experimental studies.

In conclusion, HDL are composed of subclasses of varying sizes, compositions and functions. The metabolic and inflammatory microenvironment in people with diabetes may modify the remodeling, distribution and functionalities of HDL-P. In this prospective cohort of Chinese adults with T2D, we observed an independent and inverse associations of small HDL-P with incident CVD and all-cause mortality. These HDL-P improved the risk stratification of adverse outcomes, suggesting their prognostic utility in explaining residual risk. Further investigations elucidating the vascular effects of HDL subclasses in diabetes are warranted.

## Supplementary Information

**Additional file 1: Table S1.** Intercorrelations between HDL-P measurements. **Table S2.**Concentrations of HDL-P measurements and event rates incorresponding tertile. **Table S3.**Associations of HDL-P measurements with incident CVD. **Table S4.**Associations of HDL-P measurements with all-causemortality.  As for other tables bold all* and ** to showcase stat sig variables. **Table S5.**Associations of HDL-P measurements with incident CVD estimatedby competing risk regression. **Table S6.** Associations of HDL-P measurements with incident CVD andall-cause mortality among participants with complete HDL-P measurements. **Fig. S1.**Flow chart of the study participants.

## Data Availability

The datasets used during the current study are available from the corresponding author (RCWM) upon reasonable request.
