# Supplementary material for: High-density lipoprotein subclasses and cardiovascular disease and mortality in type 2 diabetes: analysis from the Hong Kong Diabetes Biobank
Source: Cardiovasc Diabetol. 2022 Dec 31;21:293. doi: 10.1186/s12933-022-01726-y (PMC9805680; doi:10.1186/s12933-022-01726-y)
Supplement: Supplementary file 1 — Additional file 1: Table S1. Intercorrelations between HDL-P measurements. Table S2.Concentrations of HDL-P measurements and event rates incorresponding tertile. Table S3.Associations of HDL-P measurements with incident CVD. Table S4.Associations of HDL-P measurements with all-causemortality. As for other tables bold all* and ** to showcase stat sig variables. Table S5.Associations of HDL-P measurements with incident CVD estimatedby competing risk regression. Table S6. Associations of HDL-P measurements with incident CVD andall-cause mortality among participants with complete HDL-P measurements. Fig. S1.Flow chart of the study participants. [file 12933_2022_1726_MOESM1_ESM.docx]

**Additional files**

**Table S1. Intercorrelations between HDL-P measurements.**

|  | Total HDL-P | Very large HDL-P | Large HDL-P | Medium HDL-P | Small HDL-P | HDL-P size |
| --- | --- | --- | --- | --- | --- | --- |
| Total HDL-P | 1 |  |  |  |  |  |
| Very large HDL-P | 0.30 | 1 |  |  |  |  |
| Large HDL-P | 0.57 | 0.90 | 1 |  |  |  |
| Medium HDL-P | 0.93 | 0.48 | 0.75 | 1 |  |  |
| Small HDL-P | 0.77 | -0.30 | -0.08 | 0.52 | 1 |  |
| HDL-P size | 0.44 | 0.89 | 0.97 | 0.68 | -0.22 | 1 |

Derived from Pearson correlation test. All measurements were log_e_-transformed before z-scaling.

*P* values for all correlations are <0.001.

HDL-P, high-density lipoprotein particles.

**Table S2. Concentrations of HDL-P measurements and event rates in corresponding tertile.**

|  | **Incident CVD (event/N, %)**  **(125/1447, 8.6)** | | | **All-cause mortality (event/N, %)**  **(90/1991, 4.5)** | | |
| --- | --- | --- | --- | --- | --- | --- |
|  | **First tertile**  **(event/N, %)** | **Second tertile (event/N, %)** | **Third tertile (event/N, %)** | **First tertile (event/N, %)** | **Second tertile (event/N, %)** | **Third tertile (event/N, %)** |
| Total HDL-P, μmol/L | <20.47  (65/484, 13.4) | 20.47-23.05  (33/480, 6.9) | >23.05  (27/483, 5.6) | <20.03  (51/663, 7.7) | 20.03-22.75  (30/666, 4.5) | >22.75  (9/662, 1.4) |
| Very large HDL-P, ﻿μmol/L | <0.21  (22/478, 4.6) | 0.21-0.28  (51/466, 10.9) | >0.28  (52/503, 10.3) | <0.21  (13/674, 1.9) | 0.21-0.28  (21/648, 3.2) | >0.28  (56/669, 8.4) |
| Large HDL-P, ﻿μmol/L | <0.95  (44/480, 9.2) | 0.95-1.65  (48/484, 9.9) | >1.65  (33/483, 6.8) | <0.92  (22/669, 3.3) | 0.92-1.58  (25/660, 3.8) | >1.58  (43/662, 6.5) |
| Medium HDL-P, ﻿μmol/L | <4.42  (61/483, 12.6) | 4.42-5.46  (35/478, 7.3) | >5.46  (29/486, 6.0) | <4.33  (39/659, 5.9) | 4.33-5.38  (36/669, 5.4) | >5.38  (15/663, 2.3) |
| Small HDL-P, ﻿μmol/L | <14.35  (58/484, 12.0) | 14.35-15.90 (41/483, 8.5) | >15.90  (26/480, 5.4) | <14.14  (64/663, 9.7) | 14.14-15.73  (20/663, 3.0) | >15.73  (6/665, 1.0) |
| HDL-P size, nm | <9.39  (40/484, 8.3) | 9.39-9.53  (48/463, 10.4) | >9.53  (37/500, 7.4) | <9.38  (20/651, 3.1) | 9.38-9.53  (21/681, 3.1) | >9.53  (49/659, 7.4) |

CVD, cardiovascular disease; HDL-P, high-density lipoprotein particles; N, number of participants at risk.

**Table S3. Associations of HDL-P measurements with incident CVD.**

| Variable | **Model 1** | **Model 2** | **Model 3** | **Model 4** | **Model 5** | **Model 6** | **Model 7** |
| --- | --- | --- | --- | --- | --- | --- | --- |
| Total HDL-P, per SD | 0.64 (0.53, 0.76)****** | 0.69 (0.57, 0.84)****** | 0.84 (0.68, 1.03) | 0.83 (0.67, 1.02) | 0.77 (0.62, 0.95)***** | 0.76 (0.62, 0.95)***** | 0.71 (0.52, 0.97)* |
| First tertile | Reference | Reference | Reference | Reference | Reference | Reference | Reference |
| Second tertile | 0.49 (0.32, 0.74) | 0.53 (0.34, 0.80) | 0.70 (0.45, 1.08) | 0.65 (0.42, 1.02) | 0.61 (0.39, 0.96) | 0.61 (0.39, 0.96) | 0.59 (0.36, 0.96) |
| Third tertile | 0.39 (0.25, 0.60) | 0.47 (0.29, 0.75) | 0.68 (0.41, 1.13) | 0.64 (0.38, 1.08) | 0.51 (0.29, 0.89) | 0.51 (0.29, 0.90) | 0.47 (0.23, 0.95) |
|  |  |  |  |  |  |  |  |
| Very large HDL-P, per SD | 1.17 (0.96, 1.42) | 1.16 (0.95, 1.42) | 1.41 (1.13, 1.74)****** | 1.37 (1.09, 1.71)* | 1.35 (1.07, 1.70)* | 1.36 (1.07, 1.72)* | 1.44 (1.11, 1.87)** |
| First tertile | Reference | Reference | Reference | Reference | Reference | Reference | Reference |
| Second tertile | 2.47 (1.50, 4.07) | 2.56 (1.55, 4.23) | 3.12 (1.87, 5.22) | 3.14 (1.86, 5.32) | 3.04 (1.78, 5.18) | 3.15 (1.84, 5.39) | 3.06 (1.79, 5.24) |
| Third tertile | 2.36 (1.43, 3.88) | 2.41 (1.46, 3.99) | 3.12 (1.83, 5.30) | 3.02 (1.72, 5.29) | 2.81 (1.57, 5.01) | 2.97 (1.65, 5.37) | 3.23 (1.78, 5.85) |
|  |  |  |  |  |  |  |  |
| Large HDL-P, per SD | 0.91 (0.77, 1.07) | 0.91 (0.77, 1.08) | 1.40 (1.12, 1.76)****** | 1.38 (1.09, 1.75)* | 1.34 (1.05, 1.70)* | 1.36 (1.06, 1.75)* | 1.49 (1.13, 1.96)** |
| First tertile | Reference | Reference | Reference | Reference | Reference | Reference | Reference |
| Second tertile | 1.08 (0.72, 1.63) | 1.09 (0.72, 1.65) | 1.76 (1.11, 2.78) | 1.72 (1.06, 2.78) | 1.63 (1.00, 2.66) | 1.68 (1.03, 2.75) | 1.85 (1.13, 3.03) |
| Third tertile | 0.74 (0.47, 1.16) | 0.78 (0.49, 1.24) | 1.72 (0.97, 3.06) | 1.63 (0.88, 3.02) | 1.51 (0.80, 2.84) | 1.61 (0.84, 3.10) | 2.40 (1.14, 5.03) |
|  |  |  |  |  |  |  |  |
| Medium HDL-P, per SD | 0.70 (0.61, 0.81)****** | 0.72 (0.62, 0.85)****** | 0.89 (0.74, 1.08) | 0.89 (0.73, 1.09) | 0.89 (0.73, 1.09) | 0.89 (0.73, 1.09) | 0.95 (0.72, 1.23) |
| First tertile | Reference | Reference | Reference | Reference | Reference | Reference | Reference |
| Second tertile | 0.56 (0.37, 0.85) | 0.60 (0.39, 0.91) | 0.75 (0.48, 1.17) | 0.75 (0.48, 1.19) | 0.73 (0.46, 1.16) | 0.74 (0.46, 1.17) | 0.83 (0.50, 1.37) |
| Third tertile | 0.44 (0.29, 0.69) | 0.52 (0.33, 0.83) | 0.87 (0.52, 1.46) | 0.84 (0.49, 1.43) | 0.83 (0.47, 1.45) | 0.84 (0.48, 1.47) | 1.11 (0.53, 2.33) |
|  |  |  |  |  |  |  |  |
| Small HDL-P, per SD | 0.70 (0.60, 0.82)****** | 0.74 (0.62, 0.89)****** | 0.74 (0.61, 0.90)** | 0.75 (0.62, 0.91)* | 0.68 (0.55, 0.83)** | 0.66 (0.54, 0.81)** | 0.65 (0.52, 0.81)** |
| First tertile | Reference | Reference | Reference | Reference | Reference | Reference | Reference |
| Second tertile | 0.67 (0.45, 1.00) | 0.71 (0.47, 1.06) | 0.74 (0.49, 1.13) | 0.72 (0.47, 1.11) | 0.66 (0.43, 1.02) | 0.64 (0.41, 0.99) | 0.64 (0.40, 1.01) |
| Third tertile | 0.41 (0.26, 0.66) | 0.54 (0.33, 0.86) | 0.53 (0.32, 0.90) | 0.53 (0.31, 0.90) | 0.41 (0.24, 0.72) | 0.40 (0.23, 0.69) | 0.39 (0.22, 0.72) |
|  |  |  |  |  |  |  |  |
| HDL-P size, per SD | 0.91 (0.76, 1.09) | 0.93 (0.77, 1.11) | 1.24 (1.01,1.52)* | 1.23 (0.98, 1.54) | 1.30 (1.03, 1.66)* | 1.33 (1.04, 1.70)* | 1.68 (1.28, 2.22)** |
| First tertile | Reference | Reference | Reference | Reference | Reference | Reference | Reference |
| Second tertile | 1.27 (0.83, 1.93) | 1.30 (0.85, 2.00) | 1.91 (1.21, 3.02) | 1.86 (1.16, 3.01) | 1.90 (1.16, 3.11) | 1.94 (1.19, 3.18) | 2.12 (1.29, 3.48) |
| Third tertile | 0.90 (0.58, 1.41) | 0.93 (0.59, 1.47) | 1.82 (1.06, 3.15) | 1.71 (0.96, 3.06) | 1.76 (0.97, 3.22) | 1.88 (1.02, 3.49) | 2.63 (1.35, 5.12) |

Data were expressed as hazard ratio (95% confidence interval). All HDL-P measurements were log_e_-transformed before z-scaling. *, FDR <0.05; **, FDR <0.01.

Model 1: unadjusted; model 2: adjusted for age and sex; model 3: model 2+ diabetes duration, systolic blood pressure, body mass index, glycated hemoglobin, estimated glomerular filtration rate, ln (urinary albumin-creatinine ratio), and glycoprotein acetyls; model 4: model 3+oral anti-hyperglycemic drugs, insulin use, antihypertensive drugs, renin-angiotensin system blockers, and lipid-lowering drugs; model 5: model 4+low-density lipoprotein cholesterol; model 6: model 5+ln (triglycerides); model 7: model 6+high-density lipoprotein cholesterol.

**Table S4. Associations of HDL-P measurements with all-cause mortality. As for other tables bold all * and ** to showcase stat sig variables.**

| Variable | **Model 1** | **Model 2** | **Model 3** | **Model 4** | **Model 5** | **Model 6** | **Model 7** |
| --- | --- | --- | --- | --- | --- | --- | --- |
| Total HDL-P, per SD | 0.49 (0.40, 0.60)** | 0.54 (0.43, 0.67)** | 0.62 (0.49, 0.79)** | 0.62 (0.49, 0.79)** | 0.61 (0.47, 0.78)** | 0.61 (0.47, 0.78)** | 0.61 (0.43, 0.86)** |
| First tertile | Reference | Reference | Reference | Reference | Reference | Reference | Reference |
| Second tertile | 0.58 (0.37, 0.91) | 0.70 (0.44, 1.10) | 0.88 (0.55, 1.43) | 0.90 (0.55, 1.48) | 0.86 (0.52, 1.42) | 0.86 (0.52, 1.43) | 1.01 (0.58, 1.74) |
| Third tertile | 0.17 (0.08, 0.35) | 0.22 (0.11, 0.46) | 0.36 (0.17, 0.76) | 0.35 (0.16, 0.75) | 0.32 (0.15, 0.70) | 0.31 (0.14, 0.69) | 0.46 (0.18, 1.22) |
|  |  |  |  |  |  |  |  |
| Very large HDL-P, per SD | 1.54 (1.19, 1.99)** | 1.40 (1.07, 1.82)* | 1.50 (1.14, 1.97)** | 1.35 (1.03, 1.77)* | 1.39 (1.04, 1.86)* | 1.39 (1.03, 1.87)* | 1.75 (1.19, 2.58)** |
| First tertile | Reference | Reference | Reference | Reference | Reference | Reference | Reference |
| Second tertile | 1.69 (0.85, 3.38) | 1.62 (0.81, 3.25) | 1.86 (0.91, 3.78) | 1.67 (0.82, 3.43) | 1.72 (0.83, 3.55) | 1.73 (0.83, 3.59) | 1.49 (0.72, 3.11) |
| Third tertile | 4.48 (2.45, 8.20) | 3.79 (2.05, 6.99) | 4.51 (2.34, 8.70) | 3.75 (1.89, 7.45) | 3.91 (1.94, 7.89) | 3.96 (1.93, 8.10) | 4.37 (2.16, 8.84) |
|  |  |  |  |  |  |  |  |
| Large HDL-P, per SD | 1.15 (0.91, 1.45) | 1.08 (0.86, 1.37) | 1.47 (1.11, 1.94)** | 1.35 (1.01, 1.80)* | 1.36 (1.02, 1.83)* | 1.38 (1.01, 1.88)* | 1.85 (1.23, 2.76)** |
| First tertile | Reference | Reference | Reference | Reference | Reference | Reference | Reference |
| Second tertile | 1.15 (0.65, 2.04) | 1.11 (0.62, 1.97) | 1.81 (0.95, 3.42) | 1.62 (0.83, 3.16) | 1.60 (0.82, 3.13) | 1.63 (0.83, 3.21) | 1.89 (0.97, 3.68) |
| Third tertile | 2.00 (1.20, 3.35) | 1.81 (1.07, 3.06) | 3.60 (1.86, 6.96) | 3.10 (1.54, 6.23) | 3.11 (1.54, 6.29) | 3.23 (1.54, 6.76) | 7.65 (3.52, 16.65) |
|  |  |  |  |  |  |  |  |
| Medium HDL-P, per SD | 0.67 (0.58, 0.78)** | 0.67 (0.56, 0.79)** | 0.80 (0.65, 0.98)* | 0.78 (0.63, 0.95)* | 0.78 (0.64, 0.96)* | 0.77 (0.62, 0.95)* | 0.89 (0.66, 1.19) |
| First tertile | Reference | Reference | Reference | Reference | Reference | Reference | Reference |
| Second tertile | 0.91 (0.58, 1.42) | 1.01 (0.64, 1.60) | 1.24 (0.75, 2.03) | 1.28 (0.77, 2.13) | 1.25 (0.75, 2.08) | 1.21 (0.73, 2.03) | 1.64 (0.95, 2.84) |
| Third tertile | 0.37 (0.21, 0.68) | 0.43 (0.23, 0.80) | 0.73 (0.37, 1.42) | 0.73 (0.36, 1.45) | 0.73 (0.36, 1.46) | 0.69 (0.34, 1.40) | 1.66 (0.68, 4.04) |
|  |  |  |  |  |  |  |  |
| Small HDL-P, per SD | 0.51 (0.45, 0.57)** | 0.48 (0.41, 0.56)** | 0.48 (0.40, 0.58)** | 0.51 (0.43, 0.62)* | 0.47 (0.39, 0.58)** | 0.47 (0.38, 0.57)** | 0.47 (0.38, 0.59)** |
| First tertile | Reference | Reference | Reference | Reference | Reference | Reference | Reference |
| Second tertile | 0.30 (0.18, 0.50) | 0.36 (0.22, 0.59) | 0.42 (0.25, 0.71) | 0.45 (0.26, 0.76) | 0.42 (0.25, 0.71) | 0.42 (0.25, 0.72) | 0.45 (0.26, 0.79) |
| Third tertile | 0.09 (0.04, 0.21) | 0.13 (0.06, 0.31) | 0.17 (0.07, 0.41) | 0.19 (0.08, 0.45) | 0.16 (0.07, 0.39) | 0.16 (0.07, 0.39) | 0.18 (0.07, 0.46) |
|  |  |  |  |  |  |  |  |
| HDL-P size, per SD | 1.43 (1.20, 1.70)** | 1.37 (1.14, 1.65)** | 1.63 (1.33, 2.00)** | 1.54 (1.24, 1.92)** | 1.67 (1.32, 2.12)** | 1.70 (1.33, 2.16)** | 2.93 (2.22, 3.87)** |
| First tertile | Reference | Reference | Reference | Reference | Reference | Reference | Reference |
| Second tertile | 1.00 (0.54, 1.85) | 0.93 (0.50, 1.72) | 1.59 (0.82, 3.08) | 1.36 (0.69, 2.71) | 1.35 (0.68, 2.69) | 1.37 (0.69, 2.76) | 1.51 (0.76, 3.01) |
| Third tertile | 2.46 (1.46, 4.14) | 2.06 (1.20, 3.51) | 3.98 (2.08, 7.62) | 3.16 (1.60, 6.25) | 3.19 (1.61, 6.32) | 3.34 (1.63, 6.84) | 5.87 (2.84, 12.15) |

Data were expressed as hazard ratio (95% confidence interval). All HDL-P measurements were log_e_-transformed before z-scaling. *, FDR <0.05; **, FDR <0.01.

Model 1: unadjusted; model 2: adjusted for age and sex; model 3: model 2+ diabetes duration, systolic blood pressure, body mass index, glycated hemoglobin, estimated glomerular filtration rate, ln (urinary albumin-creatinine ratio), and glycoprotein acetyls; model 4: model 3+oral anti-hyperglycemic drugs, insulin use, antihypertensive drugs, renin-angiotensin system blockers, lipid-lowering drugs, and prevalent cardiovascular disease; model 5: model 4+low-density lipoprotein cholesterol; model 6: model 5+ln (triglycerides); model 7: model 6+high-density lipoprotein cholesterol.

**Table S5. Associations of HDL-P measurements with incident CVD estimated by competing risk regression.**

| Variable | **Model 1** | **Model 2** | **Model 3** | **Model 4** | **Model 5** | **Model 6** | **Model 7** |
| --- | --- | --- | --- | --- | --- | --- | --- |
| Total HDL-P, per SD | 0.65 (0.54, 0.78)** | 0.71 (0.58, 0.86)** | 0.85 (0.69, 1.03) | 0.84 (0.68, 1.03) | 0.78 (0.63, 0.96)* | 0.78 (0.63, 0.96)* | 0.72 (0.53, 0.98)* |
| Very large HDL-P, per SD | 1.15 (0.94, 1.40) | 1.14 (0.91, 1.42) | 1.37 (1.10, 1.72)* | 1.33 (1.06, 1.67)* | 1.31 (1.04, 1.66)* | 1.32 (1.05, 1.68)* | 1.39 (1.06, 1.83)* |
| Large HDL-P, per SD | 0.90 (0.78, 1.05) | 0.90 (0.77, 1.06) | 1.38 (1.11, 1.71)* | 1.36 (1.08, 1.70)* | 1.31 (1.03, 1.65)* | 1.33 (1.06, 1.68)* | 1.44 (1.11, 1.87)* |
| Medium HDL-P, per SD | 0.70 (0.61, 0.82)** | 0.73 (0.61, 0.86)** | 0.90 (0.74, 1.09) | 0.89 (0.72, 1.10) | 0.89 (0.73, 1.09) | 0.89 (0.73, 1.09) | 0.94 (0.72, 1.23) |
| Small HDL-P, per SD | 0.73 (0.63, 0.84)** | 0.77 (0.66, 0.91)** | 0.77 (0.64, 0.93)* | 0.78 (0.65, 0.94)* | 0.71 (0.59, 0.86)** | 0.70 (0.58, 0.85)** | 0.69 (0.56, 0.86)** |
| HDL-P size, per SD | 0.91 (0.76, 1.09) | 0.91 (0.76, 1.10) | 1.21 (0.98, 1.48) | 1.19 (0.96, 1.48) | 1.25 (0.99, 1.58) | 1.27 (1.01, 1.61)* | 1.54 (1.19, 1.99)** |

Data were expressed as subdistribution hazard ratio (95% confidence interval). All HDL-P measurements were log_e_-transformed before z-scaling. *, FDR <0.05; **, FDR <0.01.

Model 1: unadjusted; model 2: adjusted for age and sex; model 3: model 2+ diabetes duration, systolic blood pressure, body mass index, glycated hemoglobin, estimated glomerular filtration rate, ln (urinary albumin-creatinine ratio), and glycoprotein acetyls; model 4: model 3+oral antihyperglycemic drugs, insulin use, antihypertensive drugs, renin-angiotensin system blockers, and lipid-lowering drugs; model 5: model 4+low-density lipoprotein cholesterol; model 6: model 5+ln (triglycerides); model 7: model 6+high-density lipoprotein cholesterol.

**Table S6. Associations of HDL-P measurements with incident CVD and all-cause mortality among participants with complete HDL-P measurements.**

|  | **Model 1** | **Model 2** | **Model 3** | **Model 4** | **Model 5** | **Model 6** | **Model 7** |
| --- | --- | --- | --- | --- | --- | --- | --- |
| **Incident CVD** |  |  |  |  |  |  |  |
| Total HDL-P, per SD | 0.65 (0.54, 0.78)** | 0.69 (0.61, 0.78)** | 0.83 (0.72, 0.95)* | 0.81 (0.71, 0.94)* | 0.79 (0.68, 0.91)** | 0.78 (0.68, 0.90)** | 0.80 (0.65, 0.99)* |
| Very large HDL-P, per SD | 1.15 (0.96, 1.37) | 1.09 (0.96, 1.23) | 1.16 (1.01, 1.33) | 1.15 (0.995, 1.33) | 1.14 (0.98, 1.33) | 1.16 (0.997, 1.36) | 1.35 (1.13, 1.62)** |
| Large HDL-P, per SD | 0.84 (0.70, 0.995) | 0.88 (0.77, 0.99) | 1.06 (0.92, 1.23) | 1.05 (0.90, 1.23) | 1.04 (0.89, 1.22) | 1.08 (0.91, 1.28) | 1.39 (1.12, 1.72)** |
| Medium HDL-P, per SD | 0.66 (0.55, 0.79)** | 0.72 (0.63, 0.82)** | 0.88 (0.76, 1.01) | 0.87 (0.75, 1.00) | 0.86 (0.74, 0.99) | 0.86 (0.74, 0.997) | 1.01 (0.80, 1.27) |
| Small HDL-P, per SD | 0.72 (0.62, 0.84)** | 0.72 (0.64, 0.81)** | 0.78 (0.69, 0.89)** | 0.77 (0.68, 0.88)** | 0.73 (0.64, 0.84)** | 0.71 (0.61, 0.81)** | 0.72 (0.61, 0.84)** |
| HDL-P size, per SD | 0.89 (0.73, 1.08) | 0.94 (0.82, 1.07) | 1.10 (0.95,1.26) | 1.10 (0.95, 1.27) | 1.12 (0.95, 1.30) | 1.15 (0.98, 1.35) | 1.53 (1.26, 1.85)** |
|  |  |  |  |  |  |  |  |
| **All-cause mortality** |  |  |  |  |  |  |  |
| Total HDL-P, per SD | 0.50 (0.41, 0.61)** | 0.55 (0.44, 0.69)** | 0.63 (0.50, 0.81)** | 0.64 (0.50, 0.81)** | 0.62 (0.48, 0.80)** | 0.62 (0.48, 0.80)** | 0.63 (0.43, 0.91)* |
| Very large HDL-P, per SD | 1.76 (1.46, 2.12)** | 1.68 (1.39, 2.04)** | 1.72 (1.38, 2.14)** | 1.61 (1.27, 2.04)** | 1.72 (1.34, 2.21)** | 1.73 (1.34, 2.22)** | 2.92 (2.12, 4.04)** |
| Large HDL-P, per SD | 1.35 (1.08, 1.68)** | 1.31 (1.04, 1.65)* | 1.52 (1.17, 1.97)** | 1.42 (1.08, 1.87)* | 1.47 (1.10, 1.95)** | 1.51 (1.12, 2.03)** | 3.31 (2.17, 5.05)** |
| Medium HDL-P, per SD | 0.65 (0.53, 0.80)** | 0.69 (0.55, 0.86)** | 0.81 (0.63, 1.03) | 0.79 (0.61, 1.01) | 0.79 (0.61, 1.02) | 0.78 (0.60, 1.01) | 1.12 (0.74, 1.68) |
| Small HDL-P, per SD | 0.51 (0.45, 0.57)** | 0.48 (0.41, 0.56)** | 0.50 (0.41, 0.60)** | 0.53 (0.44, 0.64)** | 0.48 (0.39, 0.59)** | 0.47 (0.38, 0.58)** | 0.48 (0.38, 0.60)** |
| HDL-P size, per SD | 1.50 (1.27, 1.77)** | 1.46 (1.22, 1.74)** | 1.60 (1.31,1.95)** | 1.52 (1.22, 1.88)** | 1.63 (1.29, 2.05)** | 1.66 (1.32, 2.10)** | 2.87 (2.20, 3.75)** |

Data were expressed as hazard ratio (95% confidence interval). All HDL-P measurements were log_e_-transformed before z-scaling. *, FDR <0.05; **, FDR <0.01.

Model 1: unadjusted; model 2: adjusted for age and sex; model 3: model 2+ diabetes duration, systolic blood pressure, body mass index, glycated hemoglobin, estimated glomerular filtration rate, ln (urinary albumin-creatinine ratio), and glycoprotein acetyls; model 4: model 3+oral anti-hyperglycemic drugs, insulin use, antihypertensive drugs, renin-angiotensin system blockers, lipid-lowering drugs, and prevalent cardiovascular disease (for all-cause mortality); model 5: model 4+low-density lipoprotein cholesterol; model 6: model 5+ln (triglycerides); model 7: model 6+high-density lipoprotein cholesterol.

**Figure S1. Flow chart of the study participants.**


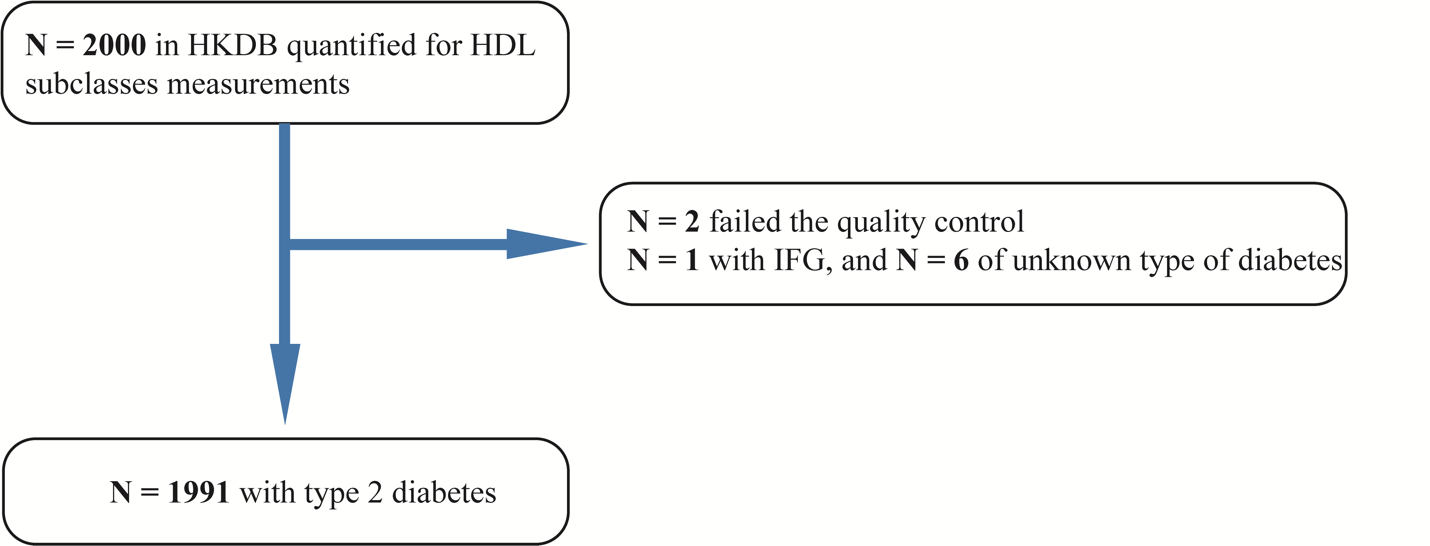


HDL, high-density lipoprotein; HKDB, Hong Kong Diabetes Biobank; IFG, impaired fasting glucose
